# Research on the Multiple Factors Influencing Human Identification Based on Pyroelectric Infrared Sensors

**DOI:** 10.3390/s18020604

**Published:** 2018-02-16

**Authors:** Junwei Yan, Ping Lou, Ruiya Li, Jianmin Hu, Ji Xiong

**Affiliations:** 1 School of Information Engineering, Wuhan University of Technology, Wuhan 430070, China; junweiyan@whut.edu.cn (J.Y.); louping@whut.edu.cn (P.L.); hujianmin@whut.edu.cn (J.H.); 2 School of Mechanical and Electronic Engineering, Wuhan University of Technology, Wuhan 430070, China; liruiya@whut.edu.cn

**Keywords:** pyroelectric infrared (PIR) sensor, identification, mapping model, macro-factor, micro-factor

## Abstract

Analysis of the multiple factors affecting human identification ability based on pyroelectric infrared technology is a complex problem. First, we examine various sensed pyroelectric waveforms of the human body thermal infrared signal and reveal a mechanism for affecting human identification. Then, we find that the mechanism is decided by the distance, human target, pyroelectric infrared (PIR) sensor, the body type, human moving velocity, signal modulation mask, and Fresnel lens. The mapping relationship between the sensed waveform and multiple influencing factors is established, and a group of mathematical models are deduced which fuse the macro factors and micro factors. Finally, the experimental results show the macro-factors indirectly affect the recognition ability of human based on the pyroelectric technology. At the same time, the correctness and effectiveness of the mathematical models is also verified, which make it easier to obtain more pyroelectric infrared information about the human body for discriminating human targets.

## 1. Introduction

In conventional biometric systems, the complex structure of certain body parts of each subject, such as a human iris, fingerprints, facial or hand geometry, are measured optically, analyzed digitally, and converted into a digital code. From the thermal perspective, each person acts as a distributed infrared source whose thermal distribution is determined by geometric shape and the thermal infrared emission from the body. The average heat a human frame radiates is about 100 W/m^2^ [1]. The temperature of a typical human body is about 37 °C (or 98 °F), and as the temperature between the human body and the environment is different, there is a heat exchange between them. Thermal infrared detectors can sense the wavelength range from 8 to 14 μm and be able to detect humans within a fairly reasonable distance. The PIR sensor can sense the thermal infrared radiation from human body at a certain distance [2,3] (2–4 m without a Fresnel lens and ~12 m with a Fresnel lens).

In recent years, with the continuous development of sensor technology, distributed inference and learning technology, human behavioral information can now be measured by passive (e.g., thermal and pressure) or active sensors (e.g., ultrasound and laser), and spatially distributed sensors nodes with computation and communication capabilities can work together to achieve complex tasks. Therefore, researchers have attempted to combine their virtues and develop distributed sensor networks based on pyroelectric infrared sensor nodes [4,5,6]. In order to identify humans, the pyroelectric infrared sensor has various advantages as follows:

- High capability in detecting infrared radiation.
- Low power consumption, and the computational complexity, communication overhead, and networking data throughput are small.
- They only can respond to a moving target. Due to their small structure, the installation environment requirement is not high.
- They are independent of illumination and have strong robustness to background color changes.
- The sensitivity range of their angular rate is about 0.1 rad/s to 3 rad/s, in a distance of less than 15 m [7].
- They can obtain better field of view (FOV) when combined with a low price Fresnel lens array. Thus, compared with traditional video systems, the use of distributed wireless pyroelectric sensor networks can provide better spatial coverage and reduce the time restrictions of system deployment [7,8,9].

PIR detectors have been widely used in people-detecting systems. In the work of Hashimoto et al., PIR detector arrays instead of camera sensors are used to detect the number of persons and their directions of movement in a wide door. By using pattern recognition methods, the movement direction detection accuracy can be 99% and for the number of passers, it can be 95% [10].

Zappi et al. built a low-cost PIR sensor-based wireless network system for detecting the direction of movement and distinguishing the number of people passing [11]. The system is composed of three sensor nodes equipped with PIR sensors having modified Fresnel lenses to narrow their fields of view. Based on the data set collected from PIR sensors while a single person and a group of people (two and three) were walking in line as well as walking side by side, they showed 100% correct detection of direction of movement and 89% correct detection of the number of people. Then they used a feature extraction and fusion algorithm to track people moving in a hallway and prove that distance can affect the human body identification [12]. As Lee presented in [13], the analog output signal of PIR sensors involves more aspects beyond simple on-off triggering, and such features have been exploited in several ways for recognizing motion direction. Yang et al. presented a novel low-cost and small-size human tracking system in a pyroelectric infrared sensor mesh network; in this network, they use a Kalman filter and particle filter algorithm to track humans to improve the tracking accuracy [14]. Luo proposed a method for abnormal activity detection with PIR sensors [15].

In recent years, more works have presented different approaches to recognize and classify humans and the number of multiple human targets using only PIR sensors. Hao et al. introduced multiple human tracking and identification with wireless distributed PIR sensor systems [16]. Zhao et al. presented a novel method to select the maximum likelihood function of Bayesian network models as the independent criterion to blindly estimate the number of moving multiple human targets [17].

Fang et al. presented a human recognition system based on a pyroelectric infrared sensor and proposed an algorithm about motion feature representation of human targets by the sensor signal spectrum. The identification process of the system includes the training with different levels of velocity and the test of various registered targets. However, the performance of this algorithm highly depends on the height of the sensor location and the distance to the target, and it is proved that the height and distance can affect the human body identification [18]. In [19], Liu designed a real-time occupancy estimation system based on a pyroelectric infrared sensor. A Hidden Markov models (HMM) is used to estimate the occupancy of a space. Compared with the traditional time window method, the proposed system with HMM can achieve higher occupancy detection accuracy.

Okuda et al. proposed a new approach to discriminate human height (adults or children) by using analog type pyroelectric sensors. Their height detection method discriminated between adults and children achieving almost 90% accuracy, so Okuda’s research proved that the human stature can also affect the recognition of the human body [20]. Ma et al. proposed a new approach to detect static thermal subjects by using an active sensor. This idea makes it possible for static thermal subjects to be detected [21].

Based on the result of previous researchers, this paper proposes a group of novel mathematical models that can qualitatively describe the factors influencing human identification. The model is composed of macro factors and micro factors. Through analysis and calculation, we reveal the mapping relation between macro factors and micro factors, and deduce the qualitative description which can affect human recognition efficiency. The experimental results show that our mathematical models reveal mapping relationships that can guide us to improve the ability of human identification.

Now, distributed pyroelectricity infrared networks equipped with lots of sensors will collect various kinds of information about changes in the user’s state and surroundings [22]. In particular, systems can analyze and interpret these collected data as a reflection of user’s behavior. At the same time, these data are used to build a rich model of the user’s context. Accordingly, this activity knowledge can help systems intelligently analyze the user’s context, and then control intelligent equipment, e.g., control light, adjust heating, etc. In addition, the PIR sensor can also be used to detect ‘no action’ events [23] and falling event [24].

This article uses the distributed pyroelectricity infrared sensor as an information collection source in a specific environment. Our system consists of a sensor module, data processing node, wireless gateway and a host computer. The sensor module consists of a pyroelectric infrared sensor, Fresnel lens and signal modulation mask. Each sensor is covered by a Fresnel lens and signal modulation mask. Once a human target moves into the sensor sensing region, the infrared radiation of the human target can be captured and transformed into an electrical signal which can be transmitted through the wireless gateway to the host computer to be further processed after preliminary noise reduction and data compression. 

However, before using a distributed wireless pyroelectric infrared sensor network in the process of detecting and identifying motion human target, we need pay attention to the following aspects:

- Different Fresnel lenses and signal modulation masks can obtain more pyroelectricity infrared information from human targets.
- Different velocity and distance which between PIR sensor and human target will have different pyroelectricity infrared information.

The goals of this paper are:

- Help researchers more easily collect and process the richer human thermal infrared information;
- Help researchers more effectively and precisely identify targets by our method;
- Help researchers more easily understand the mechanism of target identification.

In this paper, our main contributions are:

- We have developed a human thermal infrared information acquisition platform, which can collect pyroelectric infrared information about moving human targets and can effectively and precisely identify these human targets;
- The multiple factors influencing human identification based on pyroelectric infrared technology are exposed;
- We have deduced a group of mathematical models influencing the human body recognition ability, including micro and macro influence factors. Meanwhile, the mechanism of this influence on human recognition can be easily understood;
- We explained the relationship between the state of each factor and identification ability by experimental data;
- We help researchers use the macro factors to improve human identification based on the pyroelectric infrared technology.

The rest of the paper is organized as follows: in Section 2, the structure of our detector system is introduced. In Section 3, the mechanisms of macro and micro factors’ influence on human recognition are analyzed. Then the experiment results are analyzed to verify our mathematical model. Finally, the conclusions of this paper are given in Section 4.

## 2. Materials and Methods

### 2.1. Pyroelectric Data Acquisition System

**Figure 1:**
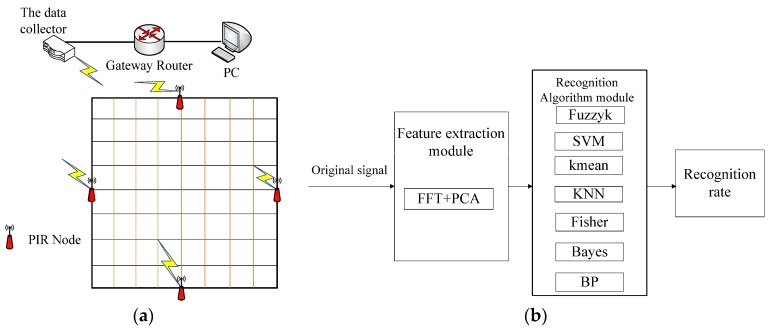
The structure of the system. (**a**) Acquisition system platform; (**b**) The structure of the algorithm.

In our system, in order to study the macro and micro influencing factors affecting human identification, reveal mapping relationships between the two kinds of factors, and accurately construct mathematical models, we designed a human body pyroelectric data acquisition system as shown in Figure 1a. In this system, the human body PIR detector module is composed of q PIR sensor, Fresnel lens, signal modulation mask and signal modulation circuit.

#### 2.1.1. PIR Sensor

The PIR sensor consists of pyroelectric materials, such as LiTiO_3_, BaTiO_3_, etc., which possesses a spontaneous polarization depending on the change of temperature. It acts like an electric capacitance and a derivative detector. The PIR sensor is suitable for working at frequencies from 0.1 to 100 Hz. As the PIR sensor responds to the rate of change of temperature, it can measure pulse type radiation power (e.g., the change rate of human thermal infrared radiation). Based on the above characteristics of the pyroelectric sensor, PIR sensors can be used for human detection and behavior understanding.

#### 2.1.2. Fresnel Lens

The Fresnel lens we employ is made of a lightweight, low-cost plastic material with good transmission characteristic in the 8~10 μm range. Concentric grooves, with increasing steepness toward the periphery of the lens, form a concave contour with optical properties, like that of a convex lens. A Fresnel lens array consists of several Fresnel lenses, curved around a detector at the focal length of the lens, covering the entire area near 0.5π FOV, and creates a set of beams on the space that have a uniform distribution. We employ the Fresnel lens array to modulate the visibility of our sensors, such that each sensor can observe events uniformly distributed over M angles.

The 8202-6 Fresnel lens is used in this paper, with a focal length is 20 mm, detection angle of 120°, and detection distance of 2–12 m. The Fresnel lens is divided two layers, the first layer has fourteen bigger lenses and the second layer has fourteen smaller lenses. The first layer is bigger than the second layer, so the first layer can sense more thermal infrared information of human body, the first layer is used to collect the thermal infrared characteristics of motions caused by upper-limb, and the second layer is used to collect the thermal infrared characteristics of motions caused by lower-limb.

#### 2.1.3. Signal Modulation Mask

Although the detection region is divided into lots of optic and dark alternate regions by the Fresnel lens, the signals are very cluttered, so we cannot effectively analyze the signals. In order to solve this problem, a kind of metal material signal modulation mask is installed in front of the Fresnel lens; the modulation mask has two categories: one is seven holes, the other is nine holes. In this way, the richer human target PIR signals will be obtained by the signal modulation mask which further optimizes the thermal infrared data of the human body, thereby improving the human targets identification ability, and also easily distinguishing between multiple human targets.

#### 2.1.4. PIR Sensor Module

**Figure 2:**
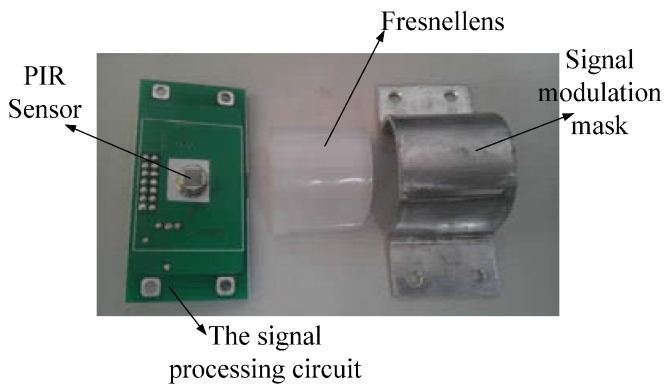
A pyroelectric infrared sensor module covered by Fresnel lens and signal modulation mask.

For its relatively stable performance, the LiTaO_3_ film pyroelectric infrared sensor is chosen as the detecting node in the system. Due to the lower receiving sensitivity of the sensor itself, every signal sensor node is covered by a Fresnel lens as shown in Figure 2. It can not only focus the infrared heat to the sensor node, but also can increase the angle and detectable distance. It was proved by some experiments that the effective detectable range can thus be increased from 2 m to 12–14 m.

#### 2.1.5. PIR Senor Node

**Figure 3:**
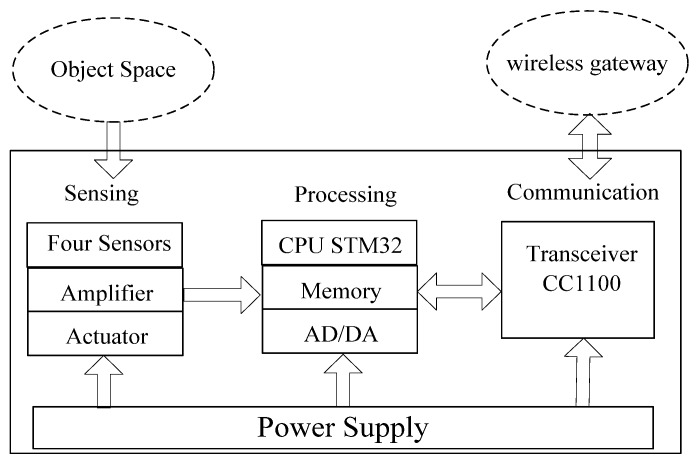
The physical PIR Senor Node device.

The PIR senor node shown in Figure 3, consists of four sensing units (PIR sensor modules), a processing unit, and a communication subsystem. The sensing unit is usually composed of a PIR sensor, amplifier and actuator. The analog original signal is captured by the sensor module and processed by the amplifier. It is converted to a digital signal by the ADC module in the processing unit equipped with a STM32 CPU, 256 kB of flash memory and 48 kB RAM. 

The memory subunit can store sensing data in a period of time. A communication subsystem interfaces the device to the network, and is composed of a transceiver subunit and processing circuit. The processed signal is sent to the wireless gateway by the communication subsystem on the basis of Zigbee protocol in some specified interval. Moreover, the whole system is powered by a power unit that is possible to support it by an energy scavenging unit like solar cells. The physical device is shown in Figure 3.

When a human target moves into the PIR sensor range, the PIR sensors can detect the thermal infrared radiation data of the human body, and convert them into electrical signals. The electrical signals are processed by the data processing unit, and then are transmitted to a data collection unit by wireless channel. Finally, we use a recognition algorithm module to analyze the thermal infrared data of human body in the computer. In Figure 1b, the structure of the algorithm can be divided into two modules: feature extraction module, and recognition algorithm module. In this paper, firstly, the characteristics of the original signal are extracted. Then, the signal is further processed to get the recognition results by classification module. Finally, we can get many kinds of recognition results, and compare these recognition results.

In the feature extraction section, the PCA [25] and the FFT [26] algorithms are used to extract pyroelectric features. In the recognition section, we can get the different recognition results through the data analysis, including FuzzyK [27], Kmean [28], KNN [29], SVM [30], Bayes [31], Fisher [32], BP [33].

In order to design the wireless network platform based on PIR sensors, we studied the design method for various video sensor platforms. We built a distributed pyroelectric infrared sensor network using several PIR sensor nodes, a wireless gateway and a host computer to detect and identify human target motion.

#### 2.1.6. Network Configuration

The data acquisition platform of human thermal infrared information consists of four nodes, gateway nodes, and a PC. Each data acquisition node consists of two thermal infrared data acquisition modules, a data processing module and a wireless transmission module. The data acquisition node can collect real-time changes in human thermal infrared information. 

Each PIR sensor node has two PIR sensor modules, which can collect human thermal infrared signals and transmit the data to the wireless gateway at 100 bps/s sampling rate. If the node transmits the data to the wireless gateway at 25,600 bps, we need at least 25.6 Kbps bandwidth, so we use the NRF24L01wireless module that has 1–2 Mbps bandwidth, and ensure the validity of the data transmission. The system has four PIR sensor nodes, so the wireless gateway needs four wireless channels. At the same time, the data transmission local area networks consist of the gateway and PC, therefore, the original data will be transmitted to the PC by the TCP/IP protocol. The data transmission protocol is made up of 40 bytes, 32 bytes of them are valid data, the frame header has 2 bytes, node ID is 1 byte, data number is 2 bytes, parity bits is 1 byte, and end of the frame has 2 bytes. The wireless transmission module can only send 20 bytes data at a time, so, first the 40 bytes of data packets will be divided into two packets by a SIM32 data processing chip, and then sent through the wireless module one by one.

### 2.2. Signal Processing

#### 2.2.1. Signal Processing Unit of the Sensor Module

**Figure 4:**
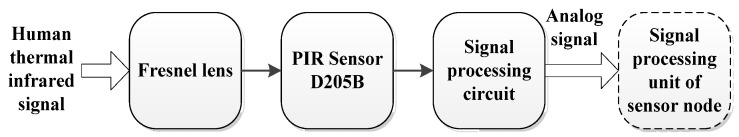
Human thermal infrared signal is processed in the sensor module.

As shown in Figure 4, the original thermal infrared signal of human target can be translated into an analog signal in three steps. 

Firstly, the original human thermal infrared signal will be focused on the pyroelectric sensor by using Fresnel lens. Then, the infrared signal is translated into a weak electrical signal by the PIR sensor. Finally, we can get the analog signal after the process of amplification and filtering of the weak electrical signals.

#### 2.2.2. Signal Processing Unit of Sensor Node

The amplitude spectrum of the sample signals can be obtained through a fast Fourier transform. The high dimensions of spectra can reduce classifier performance, so the principal component analysis (PCA) method is adopted here to a reduce the dimensions of the spectral data.

*X* are the *N* observations of *p* variable, *X =* [*x*_1_,*x*_2_,*x*_3_,…,*x_n_*]^T^, *x*_1:*n*_ is a row vector of *p* dimension which represents the spectral characteristics of each sample, *p* is the number of spectral points. The PCA algorithm steps are described as below:

(1) Standardizing the observation matrix *X* to matrix *Y*;
(2) Calculating *Z* which is the covariance matrix of *Y*;
(3) Calculating the covariance matrix eigenvalue and eigenvector of *Z*, and sorting them with values *λ*_1_ ≥ *λ*_2_ ≥ *λ*_3_ ≥ *λ*_4_ ≥…≥*λ_p_*, the corresponding eigenvectors are *U*_1_, *U*_1,_ *U*_1_,…, *U_p_*, covariance matrix *Z* can be expressed as:
  (1) $$Z=U\Lambda U^{T}$$
  where Λ is a diagonal matrix, the elements on the diagonal are eigenvalues ranked from big to small; *U* is a feature vector according to the orthogonal array composed of columns.
(4) New variable matrix with principle components as follows:
  (2) $$F=Y_{n*p}U_{p*m}$$

Each row vector of *F* matrix means a sample, its dimension dropped from *p* to *d*.

### 2.3. The Mapping Mechanism Analysis of Macro Factors and Micro Factors

There are many factors that affect human identification in a pyroelectric detection system. Based on the results of previous research, we sum up several main factors among lots of factors. At the same time, we put forward the concept of macro influence factors and micro influence factors. The macro influence factors are composed of distance between human body and PIR node (HD), body type (HS), human velocity (HV), types of Fresnel lens (F) and signal modulation mask (M). Micro influence factors are composed of the difference values of wave peak and wave valley (WR), the number of wave peaks per unit time (WN), the time of the wave peak return to the reference line (WT) and the spectrum energy in unit time (SE).

The waveform data is from thermal infrared radiation of human body, so the micro factors can affect the results of human identification. In addition, ω as the angular frequency of thermal radiation will always change in the process of human movement. When the ω changes with time, the sensed waveform will be converted to micro influence factors. Therefore, there must be some mapping relationships between the two types of influence factors. We theoretically analyze the relationship between the two types of mapping in different scenes, and construct a group of mathematical models.

#### 2.3.1. Analysis of PIR Signal Amplitude of the Radiation Source

**Figure 5:**
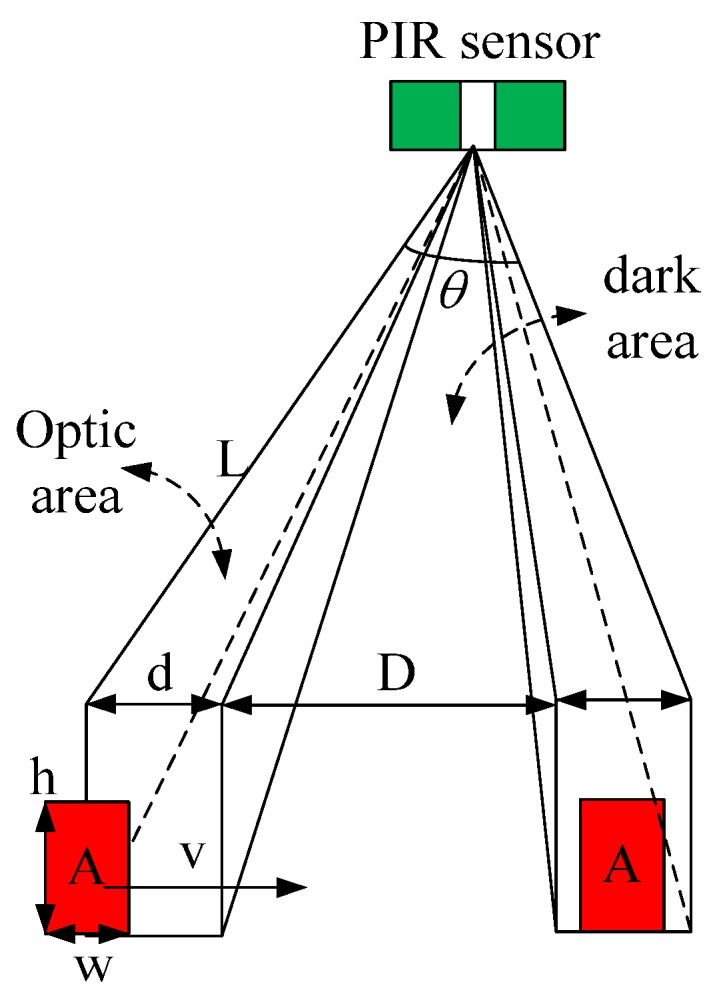
Schematic diagram of human infrared sources moving across the optic and dark region.

**Figure 6:**
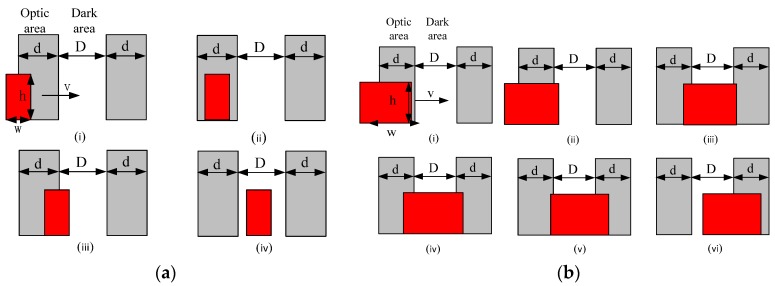
The diagrammatic sketch of human body moving across the region. (**a**) Effective width of human radiation area is smaller than the width of the optic region; (**b**) Effective width of human radiation area is larger than the width of the optic region.

In Figure 5, the human body can be approximated as a vertical cuboid radiation source, and when human comes across an optic and dark region, the sensing module will be divided into four stages as illustrated in Figure 6a. In the first stage the human radiation source walks from a dark region to an optic region. The effective radiation region will be changed. During the second stage, as effective width of human radiation area is smaller than the width of the optic region, the human will be covered in the optic region range, and effective radiation area does not change. In the third stage, the radiation source departs from the optic region. At this time, the effective radiation area of human radiation is always in change. In the fourth stage, the radiation source is in the dark region, and the effective radiation area is gone. The effective radiation area, the change area, the velocity, the height and width of radiation source, the width of optic and dark region are S_1_, ΔS_1_, v, h, w, d and D, respectively. The relationship between them is given by Equation (3).

According to the principle of the pyroelectric sensor, the output of the PIR sensor is related to varied human radiation. The effective radiation source is proportional to the change with surface area of the human body. When the surface area of the human body is larger than the optic area, the effective radiation area of human body will be complicated, and we can get the diagram of a human body coming through the optic and dark regions as shown in Figure 6b.

(1). Effective width of human radiation area is smaller than the width of the optic region:

(3) $$\begin{array}{l} S_{1}=hvt,\begin{array}{cc} & \Delta S_{1} \end{array}=hvt;\begin{array}{cc} & T_{1} \end{array}:0<t\leq w/v\\ S_{2}=hw,\begin{array}{cc} & \Delta S_{2}=0;\begin{array}{cc} & T_{2} \end{array} \end{array}:T_{1}<t\leq T_{1}+(d-w)/v\\ S_{3}=h(w-vt),\begin{array}{cc} & \Delta S_{3} \end{array}=-hvt;\begin{array}{cc} & T_{3} \end{array}:T_{2}<t\leq (T_{2}+w/v)\\ S_{4}=0,\begin{array}{cc} & \Delta S_{4}=0;\begin{array}{cc} & T_{4}:T_{3}<t\leq (T_{3}+d/v) \end{array} \end{array} \end{array}$$

**Figure 7:**
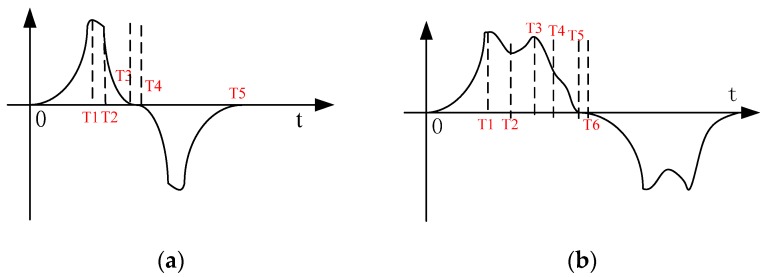
The amplitude diagrammatic of a human moving across the regions. (**a**) Effective width of human radiation area is smaller than the width of the optic region; (**b**) Effective width of human radiation area is larger than the width of the optic region.

As the amplitude of the output signal is affected by the changing thermal infrared area, the baseband output signal is determined by the optic and dark regions, resulting in that the changed amplitude will be superimposed on the baseband waveform. The waveform diagram is shown in Figure 7a.

0-T1 stands for positive half cycle, because the expanding effective area continues to increase. In T1, the waveform is in the rising stage. In T2, because the effective radiation area does not change, the inductive electrical signal will reach balance, and the wave amplitude will decrease. In T3, the effective radiation area deceases gradually, and the waveform data declines. In T4, the effective radiation area does not change, so there is no signal. T4–T5 stand for a negative half cycle which is on the time axis of symmetry. This is because we use a dual PIR sensor, so the waveform output is in the alternate polarity.

(2). Effective width of human radiation area is larger than the width of the optic region:

(4) $$\begin{array}{l} S_{1}=hvt,\begin{array}{cc} & \Delta S_{1} \end{array}=hvt;\begin{array}{cc} & T_{1} \end{array}:0<t\leq w/v\\ S_{2}=hd,\begin{array}{cc} & \Delta S_{2}=0;\begin{array}{cc} & T_{2} \end{array} \end{array}:T_{1}<t\leq T_{1}+d/v\\ S_{3}=hd+hvt,\begin{array}{cc} & \Delta S_{3} \end{array}=hvt;\begin{array}{cc} & T_{3} \end{array}:T_{2}<t\leq (T_{2}+\left(w-2d\right)/v)\\ S_{4}=h(w-d),\begin{array}{cc} & \Delta S_{4}=0;\begin{array}{cc} & T_{4}:T_{3}<t\leq (T_{3}+\left(3d-w\right)/v) \end{array} \end{array}\\ S_{5}=h(w-d)-hvt,\begin{array}{cc} & \Delta S_{5} \end{array}=-hvt;\begin{array}{cc} & T_{5} \end{array}:T_{4}<t\leq (T_{4}+\left(w-2d\right)/v)\\ S_{6}=hd,\begin{array}{cc} & \Delta S_{6}=0;\begin{array}{cc} & T_{6}:T_{5}<t\leq (T_{5}+\left(3d-w\right)/v) \end{array} \end{array} \end{array}$$

In Figure 7b it can be seen that the human body area is different, so the change in the collected energy will be different. There are some differences in the waveform details, which are reflected in the output signal. The baseband signal of the PIR sensor is decided by the PIR detector. The output signal of the PIR detector is affected by different radiation sources, which are reflected in the signal amplitude.

#### 2.3.2. Analysis of Mapping Rule

We assume that person M and person N horizontally move the same distance, the moving distance of M is $D_{x}^{M}$, the moving distance of N is $D_{x}^{N}$$D_{x}^{N}$.

The pace distance of the people is $d_{i}^{M}$ and $d_{j}^{N}$ respectively. i=1,2,3⋯m,j=1,2,3⋯n. The numbers n and m represent the number of steps. Where:

(5) $$D_{x}^{M}=D_{x}^{N}=\sum_{i}^{m}d_{i}^{M}=\sum_{j}^{n}d_{j}^{N}$$

(1). The first mapping rule

**Figure 8:**
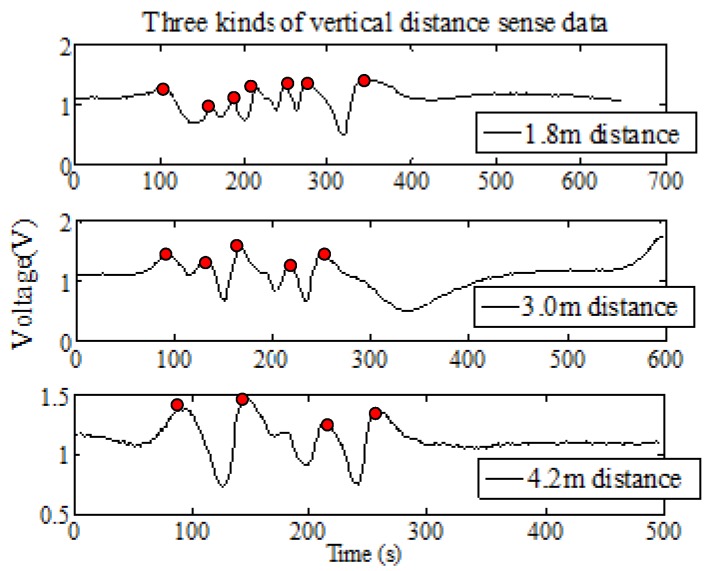
The relationship between the distance and the waveform.

In the actual application, the PIR sensor module will form an effective regular sensing region and the sensing range will be from 25°–155° (Fresnel lens is 8202-6 that called A type and the signal modulation mask has seven holes). Assuming person M and person N have a similar body type, their positions are different in the vertical distance. When they begin to walk at the same starting line and move along the same path at a constant speed, the result is that PIR sensor will generate waveform data, as shown in Figure 8.

We assume that $\theta_{i}^{M}$ and $\theta_{j}^{N}$ represent moving angle of M and N. $\omega_{i}^{M}$ and $\omega_{j}^{N}$ represent the angle change rate, so we find:

(6) $$\left\{ \begin{array}{l} \omega_{i}^{M}=\frac{\theta_{i}^{M}}{\Delta t_{i}}\\ \omega_{j}^{N}=\frac{\theta_{j}^{N}}{\Delta t_{j}} \end{array}\right.$$

When Δ*t_i_* = Δ*t_j_*, $D_{y}^{M}>D_{y}^{N}$, we can get:

(7) $$\theta_{i}^{M}<\theta_{j}^{N},\;\mathrm{so}\;\omega_{i}^{M}<\omega_{j}^{N}$$

As one can see from the above derivation, the farther the distance between the human and the PIR node is, the smaller ω is, meanwhile, the human passage through the intervals region of the alternate optic and dark regions will be less. The longer time between the wave peak and the reference line is, and the less detected peaks are. However, the smaller the distance between the human and the PIR node is, the more peaks are detected (the red point is a peak). Therefore, more information of the human thermal infrared signal can be obtained and the spectrum energy of the human is smaller.

Based on the above analysis and formula, we can get the first mapping rule: When the macro factor HD becomes smaller, there are four micro factors changing as follows:

- The micro factor WN becomes more;
- The micro factor WT gets shorter;
- The micro factor WR gets smaller;
- The micro factor SE becomes larger.

The ability of human identification will be enhanced.

(2). The second mapping rule

**Figure 9:**
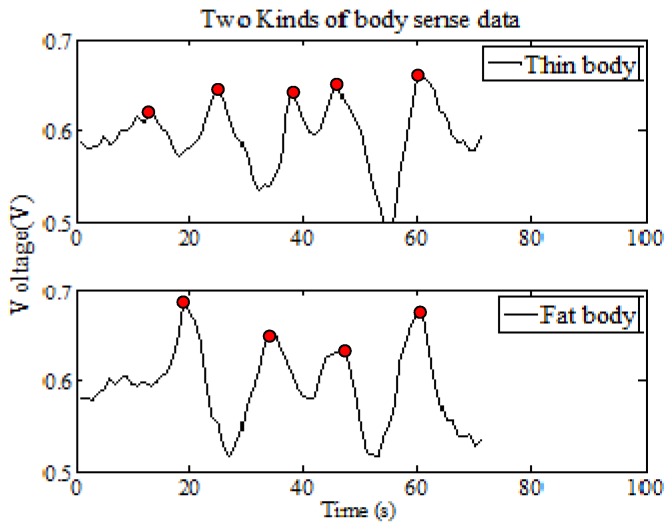
The relationship between different body and the waveform.

Suppose that two persons M and N have a larger distinction in body type, when they move at the same path and a constant speed. The PIR node module will obtain different characteristic data, as shown in Figure 9 (Fresnel lens is A type and signal modulation mask has seven holes).

**Figure 10:**
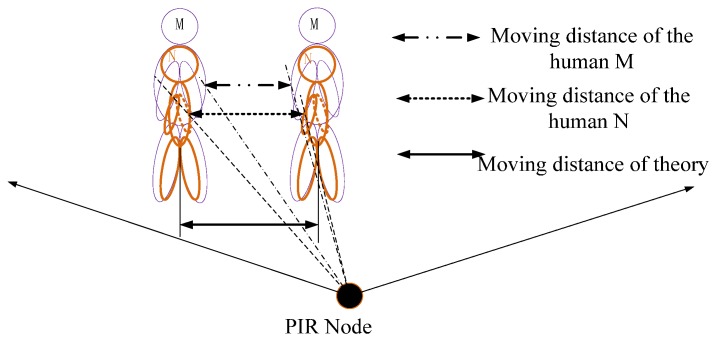
The analysis of infrared sensing for different types body.

As shown in Figure 10, the two body types are $B_{x}^{M}$ and $B_{x}^{N},$ respectively, and assuming the real movement distances of two persons are $T_{i}^{M}$ and $T_{j}^{N}$, we can get the following expression:

(8) $$\left\{ \begin{array}{l} T_{i}^{M}=d_{i}^{M}-B_{x}^{M}\\ T_{j}^{N}=d_{j}^{N}-B_{x}^{N} \end{array}\right.$$

Because Δ*t_i_* = Δ*t_j_*, $B_{x}^{M}>B_{x}^{N}$, we can get the following expression:

(9) $$\frac{T_{i}^{M}}{\Delta t_{i}}<\frac{T_{j}^{N}}{\Delta t_{j}}\Rightarrow \omega_{i}^{M}<\omega_{j}^{N}$$

Based on the above analysis and formula, we can get the second mapping rule: when the macro factor HV becomes smaller, there are four micro factors that change as follows:

- The micro factor WN becomes more;
- The micro factor WT gets shorter;
- The micro factor WR gets smaller;
- The micro factor SE becomes larger.

The human identification ability of the pyroelectric detector system will be enhanced (Fresnel lens is A-type and signal modulation mask has seven holes).

(3). The third mapping rule

**Figure 11:**
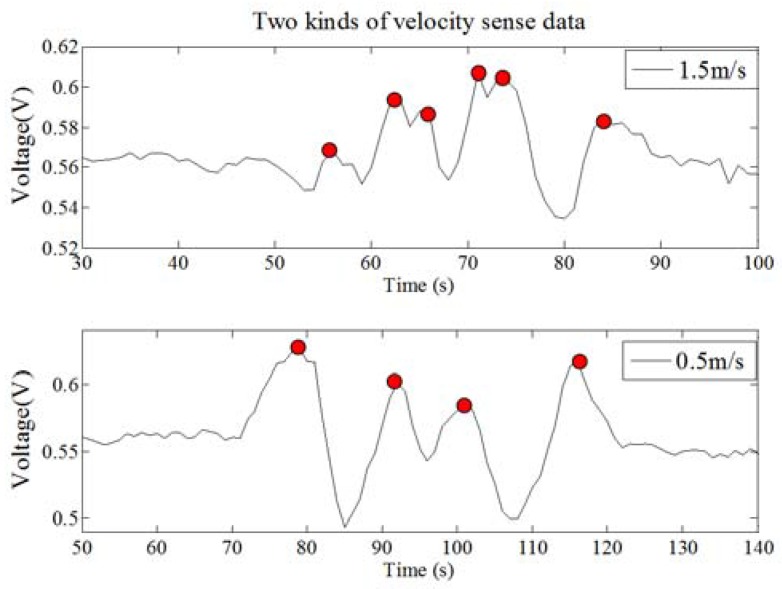
The diagram of different velocity sense waveform.

Suppose that two persons M and N have a small discrimination in their body type, and they move along the same path at a constant speed, the PIR node module will obtain different characteristic data, as shown in Figure 11.

We assume that the $V_{i}^{M}$ represents the velocity of *M*, and $V_{j}^{N}$ represents the velocity of *N*. When $D_{y}^{M}=D_{y}^{N}=0,\;V_{i}^{M}>V_{j}^{N}$ and $d_{i}^{M}=d_{j}^{N}$, we can get the expression of ω as follows:

(10) $$\left\{ \begin{array}{l} \frac{d_{i}^{M}}{V_{i}^{M}}=\frac{D_{x}^{M}}{V_{i}^{M}}=\omega_{i}^{M}\\ \frac{d_{j}^{N}}{V_{j}^{N}}=\frac{D_{x}^{N}}{V_{j}^{M}}=\omega_{j}^{N} \end{array}\right.\;\mathrm{and}\;\omega_{i}^{M}<\omega_{j}^{N}.$$

As one can see from the above derivation, a person moves faster, and the ω is larger, thewaveform changes faster, the time between the wave peak and the reference line is shorter, so the quantity of wave peaks is larger (the red point is a peak). Therefore, the more the human thermal infrared information can be contained, and the smaller the human spectrum energy is.

Based on the above analysis and formula, we can get the third mapping rule: When the macro factor HS becomes faster, there are four micro factor changes as follows:

- The micro factor WN becomes more;
- The micro factor WT gets shorter;
- The micro factor WR gets smaller;
- The micro factor SE becomes larger.

The ability of human identification will be enhanced.

(4). The fourth mapping rule

**Figure 12:**
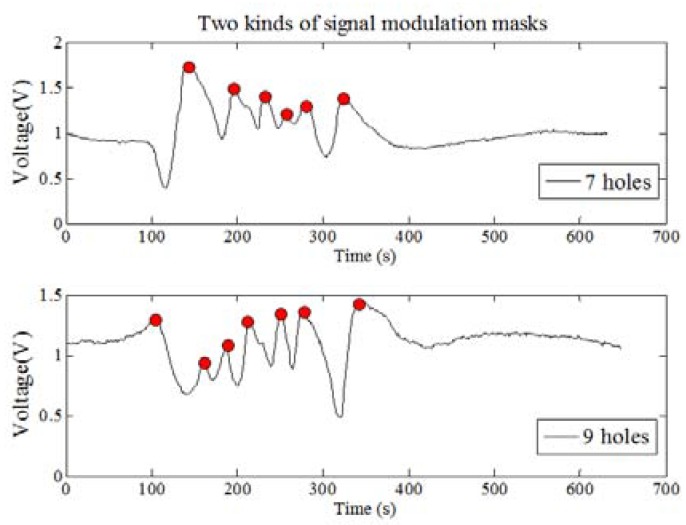
The diagram of different signal modulation mask senses waveform.

Suppose that the two persons M and N have a similar body type. They move at the same distance at a constant speed. The PIR node will obtain different characteristic data as shown in Figure 12 (Fresnel lens is A-type).

We designed a human thermal infrared detecting system which makes use of 8202-6 Fresnel lens. The upper lens has fourteen bigger lenses and the lower lens has fourteen smaller lenses. Visual area width is a, the distance between the PIR node and human target is *L*, angle range is *θ*, so we can get the following formula:

(11) $$a=\frac{2\pi L\theta }{360\times (2n-1)},\;n\;\epsilon \{W,\;V\}$$

The *n* is the number of holes and the 2*n* − 1 is the total number of optic and shade zones. The W represents that the signal modulation mask has seven holes, and the V represents nine holes.

Based on the above analysis and formula, we can get the fourth mapping rule: when the macro factor M becomes higher, there are four micro factors changes as follows:

- The micro factor WN becomes more;
- The micro factor WT gets shorter;
- The micro factor WR gets smaller;
- The micro factor SE becomes larger.

The ability of human identification will be enhanced.

#### 2.3.3. Mapping Model of Multi-Factor

Based on the above description, the pyroelectric detector system can collect different changed signals for different humans and four different conditions. We use a qualitative derivation formula, which reveals the impact of macro factors and micro factors on human identification capability. We summarize the four mapping rules between the macro and micro factors. Therefore, based on the above analysis, we can build a group of mapping mathematical models that fuse the diverse factors influencing human identification. Table 1 lists the mathematical models of the mapping relationships between the two qualitative factors. The mapping mechanism between multiple influence factors and the methods of improving human identification ability are given in Table 1.

We can find that if the macro factors change, the micro factors will be changed. The change between the micro factors and macro factors will have important effect on the human identification ability. Therefore, the two kinds of factor play an important role in application environment. The correctness and effectiveness of the model will be validated by experiments in the fourth section.

(1). Fast Fourier Transform and Principal Component Analysis (FFT+PCA) Feature Extraction

As the feature extraction algorithm directly affects the final recognition result, this paper uses normal algorithms for feature extraction. The sample signals are spectrum signals transformed by the Fast Fourier Transform. High spectrum dimensions can reduce classifier performance. The principal component analysis (PCA) method is adopted to reduce the dimensions of the spectrum data.

(2). Recognition Algorithm Type

We test and compare the seven classifiers: Naïve Bayes, support vector machines (SVM), Kmeans, Nearest Neighbor (K-NN), FuzzyK, Fisher and Bp. Classification of new instances is a lightweight task that can be implemented real-time on low-cost, low-power devices, thus allowing distributed implementation throughout the sensor network.

## 3. Results and Discussion

In this section, we will collect experimental data based on the pyroelectric detection system, which is described in the third section. We can combine with a variety of recognition algorithms to quantitatively calculate the five kinds of macro factors, such as the distance between the PIR node and human target, body types, human velocity, signal modulation mask and Fresnel lens, and analyze the relationship between the five macro factors and the methods of improving the human identification ability. In order to verify the correctness and validity of the mathematical mapping model for many influencing factors in the fourth section, we collect lots of experiment data for further analysis in this section.

### 3.1. Analysis of Experimental Datasection

The D205B infrared sensors were covered by 8202-6 Fresnel lens (A type) or 8001-1 Fresnel lens (B type) and installed in a fixed frame, which comprises the pyroelectric detector system. Then we design four experimental schemes based on the different macro factors. The first experiment is to verify distance factor, the second experiment is to verify body type factor, the third experiment is to verify human velocity factor, and the fourth experiment is to verify the signal modulation mask and Fresnel lens factors. In order to perform quantitative calculations and the comparison analyses, we define different variables and constants in the four kinds of experiments, extract the frequency spectrum value of human targets by the PCA algorithm and FFT algorithm, and then use different recognition algorithms to compute the average recognition rate, which can verify the mathematical models and mapping relationships.

In addition, in order to evaluate the influence ability of distance between the PIR node and human target, body type, human velocity, signal modulation mask and Fresnel lens factors for human identification, we use the average correct recognition rate (CRR) as a reference index, and the K fold cross validation method to evaluate the result of recognition and classification. 

There are many kinds of human body recognition methods based on pyroelectric sensor networks. The recognition performance of the identification method is evaluated by the average correct recognition rate:

CRR = (Mr/M) × 100%(12)

where Mr is the correct identification number of the samples, M is the total number of test samples.

In the actual experiments, all samples are divided into ten parts. Nine parts of all samples are used as training samples, and the remaining part is used for testing. Each experiment is repeated 10 times to get the average result. The average CRR of each kind of influencing factor will be achieved.

**Figure 13:**
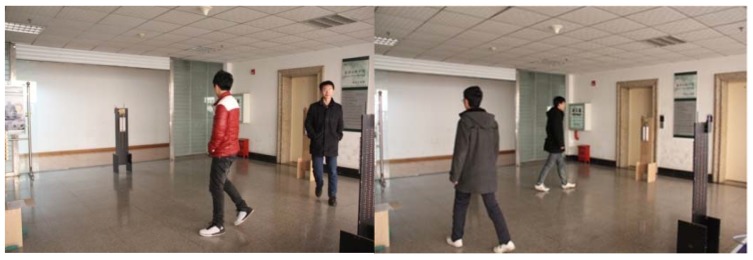
Test data collection process.

In order to obtain enough data to verify our proposed theory, 10 different persons are selected randomly as test persons. Figure 13 shows the collection of the experimental data.

#### 3.1.1. The First Experiment: Distance Factor Verification

**Figure 14:**
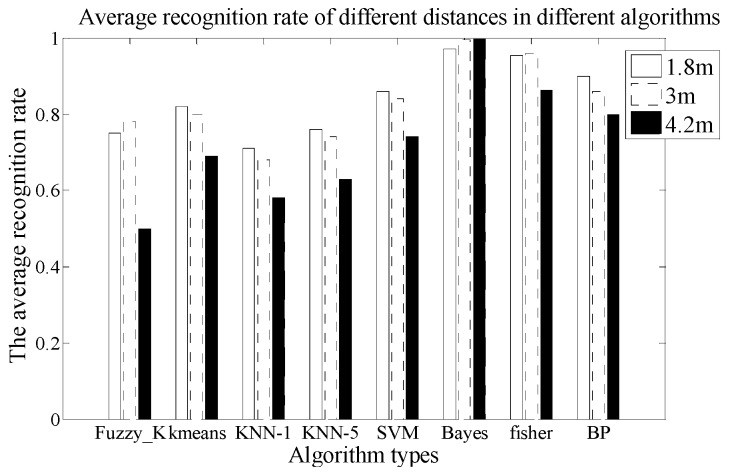
The comparison chart of average recognition rate on different distances.

In this experiment, the A type Fresnel lens is used in the PIR sensor node. Ten human targets move in three different distances between the node and the target, at a constant speed. We repeat the experiment 20 times and collect 600 groups of data. There are 200 sets of experimental data for each distance. Based on the k-fold cross method, we can use 180 sets of experimental data for training, 20 sets of experimental data for testing, and then check the computations ten times. Finally, get the average value. Then, based on the sample data, the average CRR is computed by eight kinds of algorithms. The comparison of the results is shown in Figure 14.

Comparing the recognition rate of the three paths, we can analyze that the closer the distance is, the stronger the ability of human identification is. There are five recognition algorithms, Kmeans, KNN-1, KNN-5, SVM and BP, verifying the first mapping rule in Section 4. Table 2 shows the average time consumption comparison of the different algorithms used in this experiment.

In order to illustrate the identification results of human samples in different factors. The confusion matrix under the part of algorithm is constructed. For instance, the CRR of SVM is 84% in 3 m. The confusion matrix is shown in Table 3.

#### 3.1.2. The Second Experiment: Body Type Factor Verification

In this experiment, under the same distance and velocity conditions, we verify the identification ability of the PIR detection system for different human body types. The A type of Fresnel lens and the 7-holes signal modulation mask are used in this PIR detection system. The vertical distance between the PIR sensor node and the human walking path is 3 m, and the nine test persons are asked to walk at the same speed. The experiment is repeated 20 times and the number of the samples is 180.

**Figure 15:**
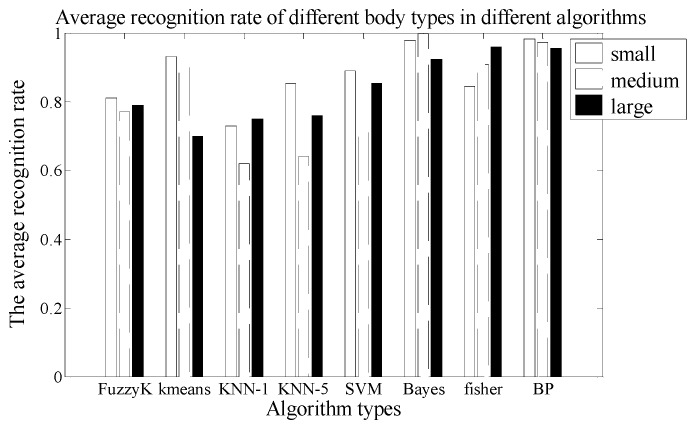
The comparison chart of average recognition rate in different body types.

There are 60 sets of experimental data for each type of human which has three persons. Based on the k-fold cross method, we can use 54 sets of experimental data for training, six sets of experimental data for testing, and then repeat ten times for checking the computations. Finally, we can get the average value. At the same time, we use eight different kinds of algorithms for human target identification and get the average recognition rate as shown in Figure 15. From the comparison results of Figure 15, we can see that the recognition ability of the PIR detection system is better for the smaller body type than for the larger body type. The second mapping rule is separately verified by FuzzyK, Kmeans, KNN-5, SVM, and BP.

Table 4 shows the average time consumption comparison of the different algorithms. As can be seen, the time consumption of the Fisher algorithm is still the shortest, while the time consumption of the BP algorithm is the longest.

A, B and C human samples are small body type, E, F and G human samples are medium body type, H, I and J human samples are large body type. The CRR of the SVM is 88% in small, 63% in medium, 86% in large. The confusion matrix is given in Table 5.

#### 3.1.3. The Third Experiment: Human Velocity Factor Verification

**Figure 16:**
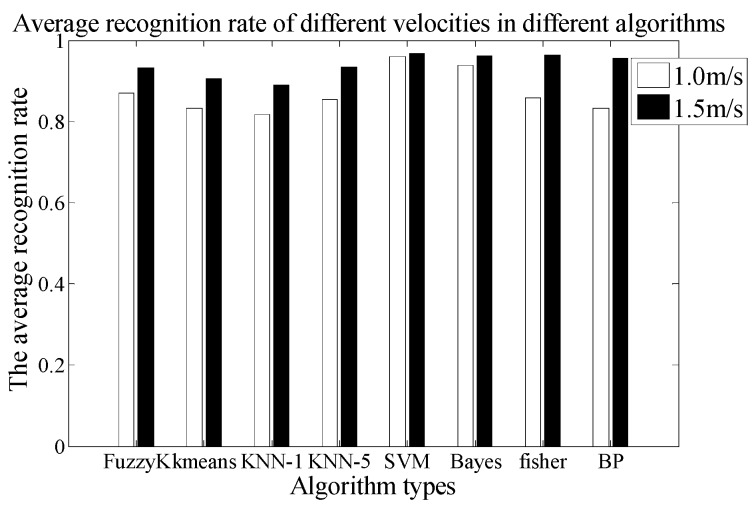
The comparison chart of average recognition rate at different velocities.

In this experiment, under the condition of the same walking path, we verify the identification ability of the PIR detection system at different walking velocities. For this test, the A type of Fresnel lens and the 7-holes signal modulation mask are used in the PIR sensor system. The vertical distance between the PIR sensor node and the human walking path is 3 m, and the ten test persons are asked to walking along the same path at two kinds of walking velocity. The experiments are repeated 10 times, and finally, 200 samples are collected. There are 100 sets of experimental data for each velocity. Based on the k-fold cross method, we can use 90 sets of experimental data for the training phase, 10 sets of experimental data for testing, and repeat ten times for checking computations. Finally, we can get the average value. At the same time, we use eight different kinds of algorithms for human target identification at different velocities and obtain the average recognition rate, which is shown in Figure 16. The recognition result of the faster human target is better than for the slower one. The third mapping rule is separately verified by FuzzyK, Kmeans, KNN-1, KNN-5, SVM, BP, Fisher and Bayes.

Table 6 shows the average time consumption comparison of the different algorithms. As can be seen, the time consumption of the Fisher algorithm is still the shortest, while the consumption of the BP algorithm is the longest.

The CRR of the SVM is 97% in 1.5 m/s. The confusion matrix is given in Table 7.

#### 3.1.4. The Fourth Experiment: Different Signal Modulation Masks and Fresnel Lenses Factors Verification

In this experiment, under the condition of same distance and velocity, we verify the identification ability of the PIR detection system for different signal modulation masks and Fresnel lenses. The ten test persons are asked to walk along three different paths. The experiments are repeated 20 times, so the number of the samples is 1200, and two different signal modulation masks are used in pyroelectric system to collect data. 

**Figure 17:**
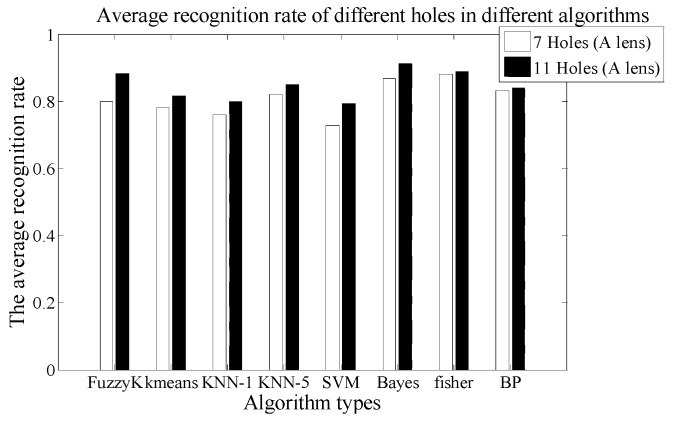
The comparison chart of average recognition rate in different mask and lens.

There are 600 sets of experimental data for each mask. Based on the k-fold cross method, 540 sets are used for training and 60 sets for testing. Then checking computations are performed ten times. Finally, we can get the average value. Then, we use eight different kinds of algorithms to compute the average CRR of ten test persons using different signal modulation masks. The results are shown in Figure 17. The more holes the signal modulation masks have, the higher the CRR is. Table 8 shows the average time consumption comparison for the different algorithms. As can be seen, the time consumption of the Fisher algorithm is still the shortest, while the consumption of the BP algorithm is the longest.

The CRR of the SVM is 78% in 11 holes. The confusion matrix is given in Table 9.

### 3.2. Analysis of the Algorithm Complexity

In Table 10, we list the complexity comparison of all algorithms in the experiment. The BP algorithm, which is the most complicated, is used for human identification, the data processing time of the BP algorithm is the longest. The complexity of Kmeans, KNN-N, SVM and Fisher is relative low. Therefore, the data processing time of these algorithms is shorter, while the Bayes and FuzzyK algorithms mainly depend on the number of iteration and the choice of the prior probability.

From the recognition rate of these algorithms, we can analyze that the recognition rate of BP algorithm is higher than the others in the four experiments, but its complexity is the highest. The recognition effect of the Bayes algorithm is the best, but the prior probability must be set up at first. Therefore, in the practical application environment and under different conditions, selecting different recognition algorithms according to the different needs will help get better results.

## 4. Conclusions

On the basis of previous research, we aimed to solve the problem of human identification ability in pyroelectric detection systems, and put forward the concept of macro influence factors and micro influence factors. With an in-depth study of the mapping mechanism between the two classes of influence factors, we firstly build the mathematical model for fusing the two classes of influence factors by the qualitative mathematical derivation that reveals the mapping relationship between the two classes of factors and their influence on human identification ability. Then, we design a pyroelectric detector system. Under different conditions, we verify the correctness and validity of the mathematical mapping model by lots of experimental data. Meanwhile, the use of eight different recognition algorithms to obtain the average CRR proves that the closer the distance, the smaller the body type, the faster human moving speed, and the more signal modulation mask holes, the easier it is for a human target to be identified in our pyroelectric detector system. Therefore, this paper builds a group of mathematical model that provides ae theoretical basis for improving human identification capability using pyroelectric detector systems.

## Figures and Tables

**Table 1:** Qualitative Mapping Relationship.

The Mapping Relation of Micro to Macro Factors	The Mapping Relation of Macro to Micro Factors
$HD\propto F（\frac{1}{FE},\frac{1}{WN},WT,WR）$	$FE\propto F(\frac{1}{HD},\frac{1}{HV},HS,M)$
$HV\propto F（\frac{1}{FE},\frac{1}{WN},WT,WR）$	$WT\propto F(\frac{1}{M},\frac{1}{HS},HD,HV)$
$HS\propto F（\frac{1}{WT},\frac{1}{WR},FE,WN）$	$WR\propto F(\frac{1}{M},\frac{1}{HS},HV,HD)$
$M\propto F（\frac{1}{WT},\frac{1}{WR},FE,WN）$	$WN\propto F(\frac{1}{HV},\frac{1}{HD},M,HS)$

*F* is Fresnel lens type coefficient.

**Table 2:** The Average Time Consumption of Different Algorithms.

Recognition Algorithm	Average Time Consumption (s)
FuzzyK	3.01
Kmeans	0.35
KNN-1	0.32
KNN-5	0.323
SVM	0.57
Bayes	1.89
Fisher	0.06
BP	8.26

**Table 3:** The Confusion Matrix of the SVM Algorithm in 3 m.

	A	B	C	D	E	F	G	H	I	J	Fault
A	15				2	1					2
B		16	2	1							1
C			19					1			
D				20							
E		1	1		15						3
F						18					2
G			2				15				3
H		1			1			17			1
I					3		2		15		
J										20	

**Table 4:** The Average Time Consumption of the Different Algorithms.

Recognition Algorithm	Average Time Consumption (s)
FuzzyK	1.48
Kmeans	0.288
KNN-1	0.244
KNN-5	0.243
SVM	0.64
Bayes	1.78
Fisher	0.052
BP	7.18

**Table 5:** The Confusion Matrix of SVM Algorithm for Small Body Type.

	A	B	C	D	E	F	G	H	I	Fault
A	17		2							1
B		19								1
C			19							1
D				12	2	3				3
E				1	15					4
F				2	2	11				5
G							20			
H							3	15		2
I									17	3

**Table 6:** The Average Time Consumption of Different Algorithms.

Recognition Algorithm	Average Time Consumption (s)
FuzzyK	2.94
Kmeans	0.49
KNN-1	0.205
KNN-5	0.55
SVM	1.3
Bayes	0.15
Fisher	0.108
BP	9.02

**Table 7:** The Confusion Matrix of the SVM Algorithm at 1.5 m/s.

	A	B	C	D	E	F	G	H	I	J	Fault
A	9										1
B		10									
C			9						1		
D				10							
E					10						
F						10					
G							10				
H								10			
I									10		
J										9	1

**Table 8:** The Average Time Consumption of the Different Algorithms.

Recognition Algorithm	Average Time Consumption (s)
FuzzyK	2.03
Kmeans	0.56
KNN-1	0.24
KNN-5	0.31
SVM	1.55
Bayes	3.0
Fisher	0.08
BP	11.7

**Table 9:** The Confusion Matrix of SVM Algorithm in 11 Holes.

	A	B	C	D	E	F	G	H	I	J	Fault
A	44				3	2					11
B		47	5			2	3				3
C			51	3		1			3		2
D		1		40		2	1				16
E					50					5	5
F	3		3	4		38					12
G			1				49	3	3		6
H				2	2		1	46			9
I	3	1							52		4
J					1			2		50	7

**Table 10:** The Complexity of the Algorithms.

Recognition Algorithm	Add	Subtraction	Multiplication	Division	Square	Most Value
FuzzyK	(2N + K + KN)L	L + LKN	(3N + K + 6KN)L	(N + K)L	(2KN)L	2KNL
Kmeans	NK	2NK	2NK	NK	NK	NK
KNN-1	NK	2NK	2NK		NK	NK
KNN-5	NK	2NK	2NK		NK	NK
SVM	N + NK	2NK	2N + 2K		NK	NK
Bayes	2NK		3NK	NK		
Fisher	17N/2	2N	11N/2	8N		
BP	7NN+N	2N+2NN	7NN + 5N	2N		

N is the number of samples; L is the number of iterations; K is the number of classifications.
